# Context-dependent release of HMGB1: cell death mode, cell type and LPS stress drive monomer and heterocomplex formation

**DOI:** 10.1186/s10020-026-01489-2

**Published:** 2026-05-02

**Authors:** Harini Pechiappan, Rebecka Heinbäck, Sigrid Stråtveit Gravning, Kjerstin Jakobsen, Richard Davies, Cecilia Aulin, Helena Erlandsson Harris

**Affiliations:** 1 Broegelmann Research Laboratory, Department of Clinical Science, University of Bergen, The Laboratory Building 5th floor, Haukeland University Hospital, Jonas Lies vei 87, Bergen, N-5021 Norway; 2 Division of Rheumatology, Department of Medicine Solna and Center for Molecular Medicine, Karolinska Institutet, Karolinska University Hospital, Stockholm, SE-171 76 Sweden

**Keywords:** HMGB1, alarmin, cancer, pyroptosis, apoptosis, necrosis

## Abstract

**Background:**

HMGB1 acts as an alarmin when released from stressed or dying cells. In vitro, HMGB1 has previously been demonstrated to readily form complexes with other molecules and through intermolecular disulfide bond formation form homodimers. Recently, dimerized HMGB1 was identified in serum of LPS-challenged mice. In cancer, HMGB1 has been described as having both tumour-promoting and tumour-suppressing features, possibly dependent on the form of HMGB1 released into the tumour microenvironment. Factors determining the form in which HMGB1 is released remain, however, largely unexplored. We therefore investigated the form of HMGB1 released during different cell death modes and in response to LPS stress using various tumour cell lines.

**Methods:**

Supernatants were collected from ten non- and LPS-treated tumour cell lines and necrotic, apoptotic and pyroptotic THP-1 monocytic cells, to assess active and passive secretion of HMGB1. Released proteins were concentrated by TCA-precipitation and analysed by Western blotting under reducing and non-reducing conditions to detect monomeric, dimeric, or HMGB1-protein complexes. Co-immunoprecipitation and LC-MS/MS were used to identify binding partners of extracellular HMGB1.

**Results:**

Tumour cells were found to release monomeric HMGB1 and HMGB1 heterocomplexes in the 50–60 kDa range, as indicated by their persistent high molecular weight under reducing conditions. Furthermore, we identified that HMGB1 interacts with ribosomal proteins, histone H2B, and SRP9 following LPS treatment of microglial SIM-A9 cells.

**Conclusions:**

HMGB1 readily formed heterocomplexes, but not homodimers in vitro across multiple cell lines, with differences between LPS-treated and untreated conditions. The form of released HMGB1 was influenced by cell type, cell death mode, and LPS stress.

**Supplementary Information:**

The online version contains supplementary material available at 10.1186/s10020-026-01489-2.

## Introduction

The nuclear protein High mobility group box 1 (HMGB1) is released into the extracellular space upon cell activation, cell stress, or cell death (Gardella et al. 2002, Lamkanfi et al. 2010, Lu et al. 2014, Scaffidi et al. 2002), from where it acts as an alarmin (Lotze and Tracey 2005, Wang et al. 1999). As an alarmin, HMGB1 is increasingly recognized as a contributor to disease pathogenesis, and circulating HMGB1 has been proposed as a clinical biomarker in cancer patients (Cheng et al. 2008, Lee et al. 2012, Shang et al. 2009). In cancer, extracellular HMGB1 has been reported to exhibit both pro- and anti-tumour activities in the tumour microenvironment (Kang et al. 2013, Xue et al. 2021, Hubert et al. 2021).

The diverse effects of extracellular HMGB1 are attributed to the molecule’s structure, including its distinct A box and B box domains and redox-sensitive cysteine residues (C23, C45, C106) (Vezzoli et al. 2011, Venereau et al. 2012, Salo et al. 2021). The redox state of HMGB1 influences its interaction with a range of immune receptors (Venereau et al. 2012, Yang et al. 2013, Yang et al. 2015), determining whether it promotes inflammation, drives chemotaxis, or being inactive. Disulfide HMGB1 (C23-C45, C106) activates TLR4/MD-2 to induce cytokine production, while the fully reduced form forms heterocomplexes with C-X-C motif chemokine ligand 12 (CXCL12) and mediates chemotactic signaling (Yang et al. 2015, Schiraldi et al. 2012, Yang et al. 2010). In addition to CXCL12, HMGB1 in vitro co-incubations has been demonstrated to form heterocomplexes with deoxyribonucleic acid (DNA), lipopolysaccharide (LPS), and Interleukin-1 beta (IL-1β), and to enhance the responses to these ligands in cell cultures (Ivanov et al. 2007, Sha et al. 2008, Youn et al. 2008, Hreggvidsdóttir et al. 2012). In cancer, the redox state of extracellular HMGB1, as well as potential heterocomplex formations, are possible explanations for the diverse effects described. HMGB1 has been demonstrated to bind TIM-3 on dendritic cells and to engage the CD24-Siglec-10 pathway on macrophages to suppress anti-tumor immunity (Chiba et al. 2012, Chen et al. 2009, Barkal et al. 2019). Additionally, HMGB1 has been shown to bind RAGE on tumour cells, activating MAPK signaling, promoting tumour proliferation, migration, and angiogenesis (Fan et al. 2024).

In addition to the well-studied redox isoforms, in vitro studies have shown that HMGB1 can self-associate, forming dimers. Monomeric HMGB1 was demonstrated to undergo oxidation-induced self-dimerization, mediated by disulfide bond formation between C106 residues of two molecules (Kwak et al. 2021). This homodimeric form has been reported to serve distinct biological functions, including protection against ROS-induced DNA damage (Kwak et al. 2021) and enhancement of pro-inflammatory signals (Kwak et al. 2025). Surface plasmon resonance studies have shown that HMGB1 self-association is highly sensitive to pH, charge density, redox state, and the presence of metal cations (Anggayasti et al. 2016, Anggayasti et al. 2016). The dimerization and oligomerization of HMGB1 are far less studied than its other redox forms.

Previous studies using mouse models of pulmonary arterial hypertension and sepsis have indicated that dimeric HMGB1 is released in a cell-type-specific manner and is influenced by factors such as mitochondrial oxidative activity and reactive oxygen species (Kwak et al. 2021, Zemskova et al. 2020). These observations suggest that both the cellular context and the mode of cell death contribute to the redox state and oligomerization of extracellular HMGB1 in vivo.

As the form of extracellular HMGB1 critically impacts the cellular response, understanding factors that influence HMGB1 release and determining its extracellular forms are crucial for interpreting its immunological effects. However, systematic characterization of HMGB1 forms released across different cell types and stress conditions remains limited. The aim of this study was to investigate how cellular stress induced by LPS and different forms of cell death influence the form and oligomerization state of released HMGB1. We examined these factors in multiple tumour cell lines known to express high levels of HMGB1 (Kang et al. 2013, Jube et al. 2012). Under the conditions examined, extracellular HMGB1 was detected as a monomer or in heterocomplexes, but not as dimers. This demonstrates that the cellular context modulates HMGB1’s extracellular forms, which may contribute to its diverse functional roles in cancer and in other pathologies.

## Materials and methods

### In vitro dimerization of HMGB1

To confirm previous reports that HMGB1 dimerizes under oxidative conditions, recombinant HMGB1 (rHMGB1, 2 µg), produced in house as previously described (Yang et al. 2010), was incubated with hydrogen peroxide (H_2_O_2_) (5, 50, 100 and 250 µM) (Merck) or copper chloride (Cu(II)Cl_2_) (1, 10, and 25 µM) (Alfa Aesar) for 2 h at 37 °C. To assess reversal of oxidation, dithiothreitol (DTT) (100 mM) (Sigma Aldrich) was added to one sample of rHMGB1 previously treated with 25 µM Cu (II)Cl₂ and 250 µM H_2_O_2_. All samples were boiled in Laemmli sample buffer (Bio-Rad Laboratories) for 5 min and subsequently separated by 4–20% polyacrylamide gel electrophoresis (Bio-Rad Laboratories) and visualized using Coomassie blue staining (Thermo Fisher Scientific).

### Cell culture

To investigate how cell type affects the formation of HMGB1 complexes and dimers, we utilized ten cell lines: SIM-A9 (microglia), three myeloid cell lines U937, THP-1 (monocytic leukemias), RAW 264.7 (murine macrophages), and six diverse tumor cell lines Nalm6 (B-cell leukemia), SK-MEL-28 (melanoma), SH-SY5Y (neuroblastoma), HeLa (cervical carcinoma), HCT-116 and Caco-2 (colorectal carcinoma). U937, Nalm6, and THP-1 were cultured in RPMI (Sigma Aldrich); RAW 264.7, HeLa, HCT-116, SK-MEL-28 and Caco-2 in DMEM (Sigma Aldrich); and SIM-A9 and SH-SY5Y in DMEM: F12 media (Gibco). HEK293T cells were cultured in DMEM (Sigma Aldrich) supplemented with 10% fetal calf serum. All culture media was supplemented with 100 U/mL penicillin, 100 µg/ml streptomycin (Gibco), and 10% fetal bovine serum (FBS) (Gibco), except for SIM-A9, where 5% horse serum was used in addition to FBS (Gibco) (see Supplementary Table 1).

All cell lines were obtained from ATCC (Manassas, VA, USA) (see Supplementary Table 1). Cells were cultured in a humidified atmosphere at 37 °C with 5% CO_2_ and passaged as directed by ATCC, for a maximum of 20 passages.

### Immunocytochemistry, confocal imaging and quantification

Immunofluorescence was used to confirm the presence of HMGB1 and changes in subcellular localization following LPS treatment. 3 × 10^4^ cells were seeded into each well of an 8-well Nunc™ Lab-Tek™ II Chamber Slide™ Systems (Thermo Fisher Scientific, Cat. No. 154534). For suspension cells, gelatin-coated slides were used to facilitate cell adherence. Cells were treated with either PBS or LPS (Sigma-Aldrich, #L6529) at 1 µg/ml or 10 µg/ml (the latter concentration for Caco-2, based on literature) (Guo et al. 2015, Wei et al. 2022) for 24 h at 37 °C. Cells were washed with phosphate-buffered saline (PBS) and fixed with 4% paraformaldehyde for 15 min at room temperature (RT). After fixation, cells were washed three times with PBS containing 0.1% Tween-20 (PBS-T), permeabilized with 0.2% Triton X-100 in PBS (15 min, RT), and washed three times again with PBS-T.

To block nonspecific binding, cells were incubated with 10% normal goat serum (DAKO) in PBS for 1 h at RT. Cells were incubated overnight at 4 °C with an anti-HMGB1 antibody (Abcam, #ab79823, 1:500) diluted in PBS-T supplemented with 0.5% normal goat serum. Cells were then rinsed three times with PBS-T and incubated with an Alexa Fluor 488-conjugated goat anti-rabbit secondary antibody (Thermo Fisher Scientific, #A-11034, 1:500) and Alexa Fluor 633-conjugated phalloidin (Thermo Fisher Scientific, #A22284, 1:40), diluted in PBS-T with 0.5% normal goat serum, for 45 min at RT in the dark.

Nuclei were counterstained with DAPI for 5 min prior to washing with PBS. Slides were mounted using ProLong™ Antifade Mountant (Thermo Fisher Scientific, Cat. No. P36935) and covered with coverslips. Imaging was performed using a Zeiss LSM800 confocal laser scanning microscope equipped with a 63×/1.4 NA oil immersion objective. Images were obtained using ZEN 2.3 SP1 FP3 (black). Pearson’s correlation coefficients between HMGB1 and DAPI stainings (per cell) were obtained using pixel-by-pixel correlation in Cell Profiler (4.1.3).

### Induction of cell death

Apoptosis, pyroptosis, or necrosis were induced in THP-1 cells to assess the passive release of HMGB1. Necrosis was induced by subjecting 1 × 10^6^ cells in Opti-MEM (Gibco) to three freeze-thaw cycles using a dry ice/EtOH mixture and a 37 °C water bath. Cells were centrifuged at 21,000 g for 10 min at 4 °C to remove cellular debris, and supernatants were collected.

To induce apoptosis, 2 × 10^5^ cells were treated with either 2.5 µM etoposide (Sigma Aldrich), 1 µM staurosporine (STS, Alfa Aesar), or DMSO as a negative control. Cells were treated for 1, 3, 6, 8, or 24 h at 37 °C, 5% CO_2_. To induce pyroptosis, 2 × 10^5^ THP-1 cells were differentiated into macrophage-like cells by treatment with 160 nM phorbol-12-myristate-13-acetate (PMA, Sigma) for 16 h. The cells were then washed twice with PBS and rested in growth media for 72 h. Cells were washed, primed with 10 ng/ml LPS (Sigma-Aldrich, #L6529, Batch no. 1245051) for 6 h, and treated with 10 µM nigericin (Invivogen) for an additional 1.25 h in Opti-MEM (Gibco). Untreated differentiated cells were used as a negative control. Cell supernatants were collected following centrifugation at 400 g for 5 minutes at 4 °C. Supernatants from untreated cells were used as negative controls for apoptosis and pyroptosis.

### LDH release and Caspase3/7 activity assays

Cell death with membrane rupture was estimated by measuring LDH release in the supernatants using Cytotoxicity Detection Kit (Roche Diagnostics, Cat. No. 11644793001) according to manufacturer’s instructions. Apoptosis was estimated by caspase 3 and 7 activity using the Caspase-Glo 3/7 Assay (Promega) according to manufacturer’s instructions. LDH absorbance was measured at 490 nm (reference: 650 nm), while caspase 3/7 activity was measured by luminescence, both using a Synergy H1 Multimode microplate reader (BioTek).

### ELISA

To confirm pyroptosis, IL-1β and TNF-α concentrations in cell supernatants were assessed by sandwich ELISA using DuoSet ELISA kits (R&D Systems). Assays were performed according to the manufacturer’s instructions. Absorbance was measured using a Synergy H1 Multimode microplate reader at 450 nm with reference at 540/560 nm. MyAssays (https://www.myassays.com/index.html), an online analysis tool, was used to determine cytokine concentrations, and the standard curve was fitted using a four-parameter logistic curve (4-PL) (Ltd. M. 2012).

### Induction of active HMGB1 secretion by LPS

To assess active release of HMGB1, 3 × 10^5^ adherent or 1 × 10^6^ suspension cells were seeded in T-75 flasks and rested overnight. Prior to stimulation, the media was replaced with Opti-MEM (Gibco). To induce HMGB1 secretion, cells were stimulated with LPS (1 µg/ml or 10 µg/ml for Caco-2) or PBS for 24 h. Cell supernatants were collected following centrifugation (400 g, 5 minutes, 4 °C). Supernatants were immediately used for protein precipitation and Western blot against HMGB1.

### Precipitation of proteins and Western blotting

Proteins in cell supernatants were precipitated using trichloroacetic acid (TCA). Briefly, proteins were precipitated by adding ice-cold TCA to a final concentration of 4%. Following gentle mixing, supernatants were incubated on ice for 30 min and centrifuged at 4700 g for 20 min at 4 °C. Pellets were washed five times with ice-cold acetone. Finally, the pellet was air-dried and resuspended in PBS. Western blot was used to detect HMGB1 in precipitated supernatants. A total of 1.5 µg protein to assess HMGB1 released during cell death and 3 µg protein for active release were loaded for each sample. Proteins were separated by electrophoresis using a 4–20% gel (Bio-Rad Laboratories), with samples treated with or without the reducing agent, tris(2-carboxyethyl) phosphine (TCEP) (1:10) (Thermo Fischer Scientific). 25 ng rHMGB1 was used as a positive control for released HMGB1. Proteins were transferred onto a 0.2 μm nitrocellulose membrane (Bio-Rad) by a wet transfer blot system (100 V, 1 h). The membranes were blocked with 5% non-fat dry milk (Cell Signaling Technology) for 1 h at RT and then incubated with 1 µg/ml anti-HMGB1 monoclonal mouse IgG2b antibody (2G7, in-house production from Karolinska Institutet) (Schierbeck et al. 2011) diluted in 1% milk-TBST overnight at 4 °C. Membranes were washed and incubated with HRP-conjugated anti-mouse IgG (Cell Signaling Technology, #7076, 1:2000) in 1% milk-TBST for 1 h at RT. Membranes were washed, and signal was detected using the Clarity Western ECL detection kit (Bio-Rad) and imaged with an iBright CL1000 (Thermo Fischer Scientific). The relative intensity of the bands was calculated using Image Lab software version 6.1 and normalized based on the intensity of the total rHMGB1 signal.

### Cloning of pCW57.1-HMGB1 and pCW57.1-p3xFlag-HMGB1

The coding sequence of HMGB1 and Flag-HMGB1 was amplified using CMV-26-p3xFlag-HMGB1 as a template. The following primers with added NheI and SalI restriction sites at the 5’ and 3’ end, respectively, were used:

GCTG*GCTAGC*CACCATGGACTACAAAGACCATGACGGTG (5’ for Flag-HMGB1), GCTG*GCTAGC*CACCATGGGCAAAGGAGATCCTAAGAAGCCG (5’ for non-tagged.

HMGB1) and CAACAA*GTCGAC*TTATTCATCATCATCATCTTCTTCTTCATCTTC (3’ for both constructs). PCR reaction was initiated with denaturation (95 °C, 5 min), followed by 30 cycles of denaturation (95 °C, 15 s), annealing (60 °C, 15 s) and elongation (72 °C, 1 min). The PCR products were thereafter inserted into pCW57.1 (Addgene, #41393) at the restriction sites of NheI and SalI.

### Lentiviral packaging and viral transduction of SIM-A9 cells

A total of 7 × 10^5^ HEK293T cells were seeded per well in a flat-bottom 6-well plate. Mixtures of 100 µl Opti-MEM™ I Reduced Serum Medium (Thermofisher Scientific), 9 µl X-tremeGENE™ 9 DNA Transfection reagent (Sigma Aldrich), 1.5 µg pCW57.1- HMGB1 or pCW57.1-p3xFlag-HMGB1, 1 µg psPAX2 (Addgene, #12260) and 0.75 µg pMD2.G (Addgene, #12259) were incubated for 20 min at RT and added dropwise to the cells, and cells were incubated overnight before standard growth media change. Thereafter, viral packaging was conducted for 72 h before harvesting by centrifugation at 500 g for 5 minutes. 7.5 × 10^5^ SIM-A9 cells were seeded per well in a 6-well plate and infected with harvested virus. Transduction efficiency was enhanced by centrifugal inoculation at 1400 g for 1 h in the presence of polybrene (2.5 µg/ml, Sigma Aldrich). Transduced cells were selected by the addition of puromycin (2.5 µg/ml, Thermofisher Scientific) to the cell medium.

### Co-immunoprecipitation and quantitative liquid chromatography with tandem mass spectrometry (LC-MS/MS)

To identify HMGB1 binding partners, transduced SIM-A9 cells were grown as previously mentioned. Non-tagged HMGB1 and p3xFlag-HMGB1 (details follow) expression was induced with 2 µg/ml doxycycline for 24 h. Growth media was then removed, and the cells incubated for 24 h in Opti-MEM supplemented with 2 µg/ml doxycycline, with or without 1 µg/ml LPS. Supernatants were collected following centrifugation at 300 g, 5 min, and p3xFlag-HMGB1 was immunoprecipitated using mouse anti-FLAG^®^ M2 magnetic beads (Millipore) according to the manufacturer’s instructions. Briefly, SIM-A9 cell supernatants and an Opti-MEM media-only control were incubated with magnetic beads at 4 °C overnight with end-to-end rotation. The beads were washed 5 times with Opti-MEM in a magnetic rack, followed by incubation with elution buffer (4% SDS, 0.1 M Tris pH 7.6, 10 mM chloroacetamide, 5 mM TCEP) at 95 °C for 5 min to denature and release bead-bound proteins. Beads were then pelleted by centrifugation at 10,000 g for 1 min, and the eluates were collected, snap frozen, and stored at − 80 °C for LC-MS/MS and Western blotting. The experiment was conducted in triplicate except for the Opti-MEM control, where a single replicate was used as a bead control.

LC-MS/MS was conducted at the Proteomics Unit at the University of Bergen. The elutions were treated with 5 mM TCEP to reduce disulfide bonds, and cysteine residues were alkylated with 10 mM chloroacetamide to prevent disulfide bond reformation. After cooling, samples were digested following the SP3 sample preparation protocol as previously described (Szigetvari et al. 2023). The DIA raw files were searched using Spectronaut (Biognosys) in direct DIA mode. Trypsin was selected as the digestion enzyme for the pulsar search. Carbamidomethylation was selected as a fixed modification, and methionine oxidation and N-terminal acetylation were selected as variable modifications. For quantitation, MS2 was chosen as the quantity MS-level, and data imputation was disabled. The file was then searched against the mouse database downloaded from Swissprot on 24 May 2024 and the common contaminant database downloaded in March 2022 (Consortium 2024). The data was processed using the Perseus package (Tyanova et al. 2016), background of measurements were removed by subtracting the Opti-MEM media-only control, followed by log2-transformation. To decrease false positives, identified proteins consisting of less than three unique peptides and not found in all replicates of at least one triplicate, were omitted from the analysis, resulting in 853 of the 2187 identified proteins used for statistical comparisons.

### Statistical analysis

LC-MS/MS statistical analysis was performed using the Perseus package, and figures were produced in GraphPad Prism version 9. Two-sample t-tests were performed on background-corrected log₂-transformed relative values, and multiple testing was adjusted using the Benjamini–Hochberg method (q ≤ 0.05 considered significant). Normality of all datasets was assessed using the Shapiro–Wilk test prior to performing parametric analyses. When data did not meet normality assumptions, non-parametric tests were applied, including the Mann–Whitney U test for two-group comparisons and the Kruskal–Wallis test with Dunn’s multiple comparisons correction for analyses involving more than two groups. All other statistical analyses were performed in GraphPad Prism. Two-way ANOVA with Dunnett’s multiple comparisons test was used for normally distributed assay datasets. *p* ≤ 0.05 was considered statistically significant.

## Results

### In vitro dimerization of HMGB1 is dependent on disulfide bridge formation

**Fig. 1:**
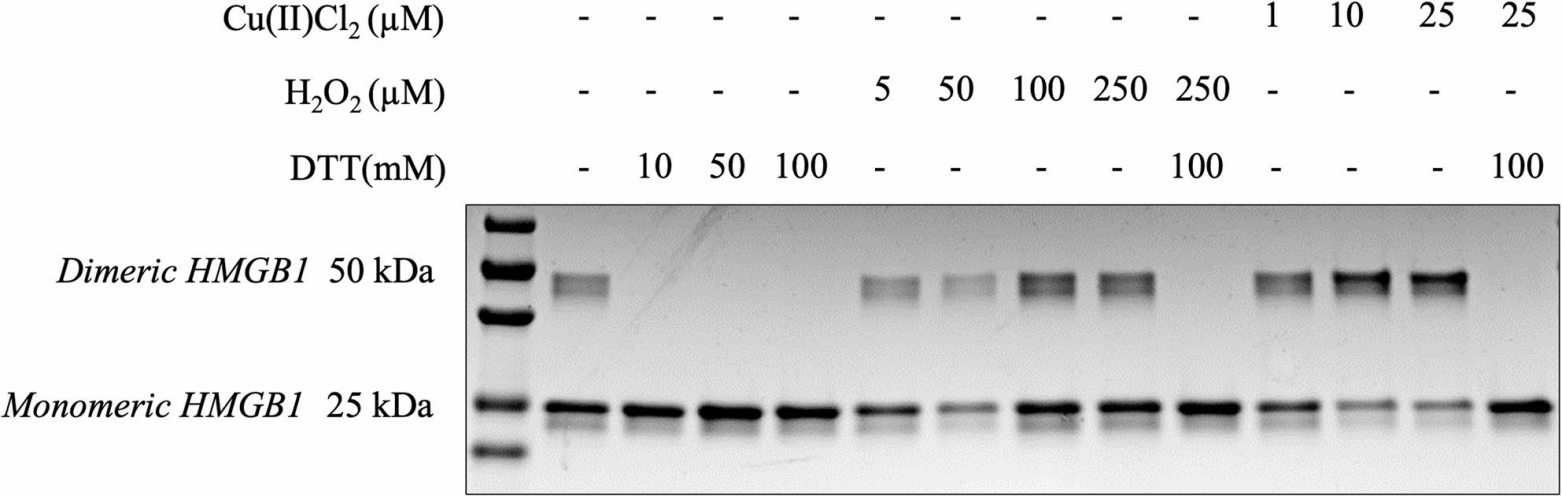
Recombinant HMGB1 dimerizes in oxidative environments. 2 µg rHMGB1 was treated with increasing concentrations of Cu(II)Cl_2_, H_2_O_2,_ and with DTT. SDS-PAGE followed by Coomassie blue staining was performed to visualize proteins in the gel

Confirming previous reports that HMGB1 dimerizes under oxidative conditions (Kwak et al. 2021), rHMGB1 was treated with varying concentrations of Cu(II)Cl_2_ and H_2_O_2_. Following oxidation, HMGB1 dimerized (50 kDa), and dimerization was reversed by the addition of the reducing agent DTT, confirming the dependency on disulfide bridges (Fig. 1).

### LPS-induced cytoplasmic translocation of HMGB1 is cell-type dependent

**Fig. 2:**
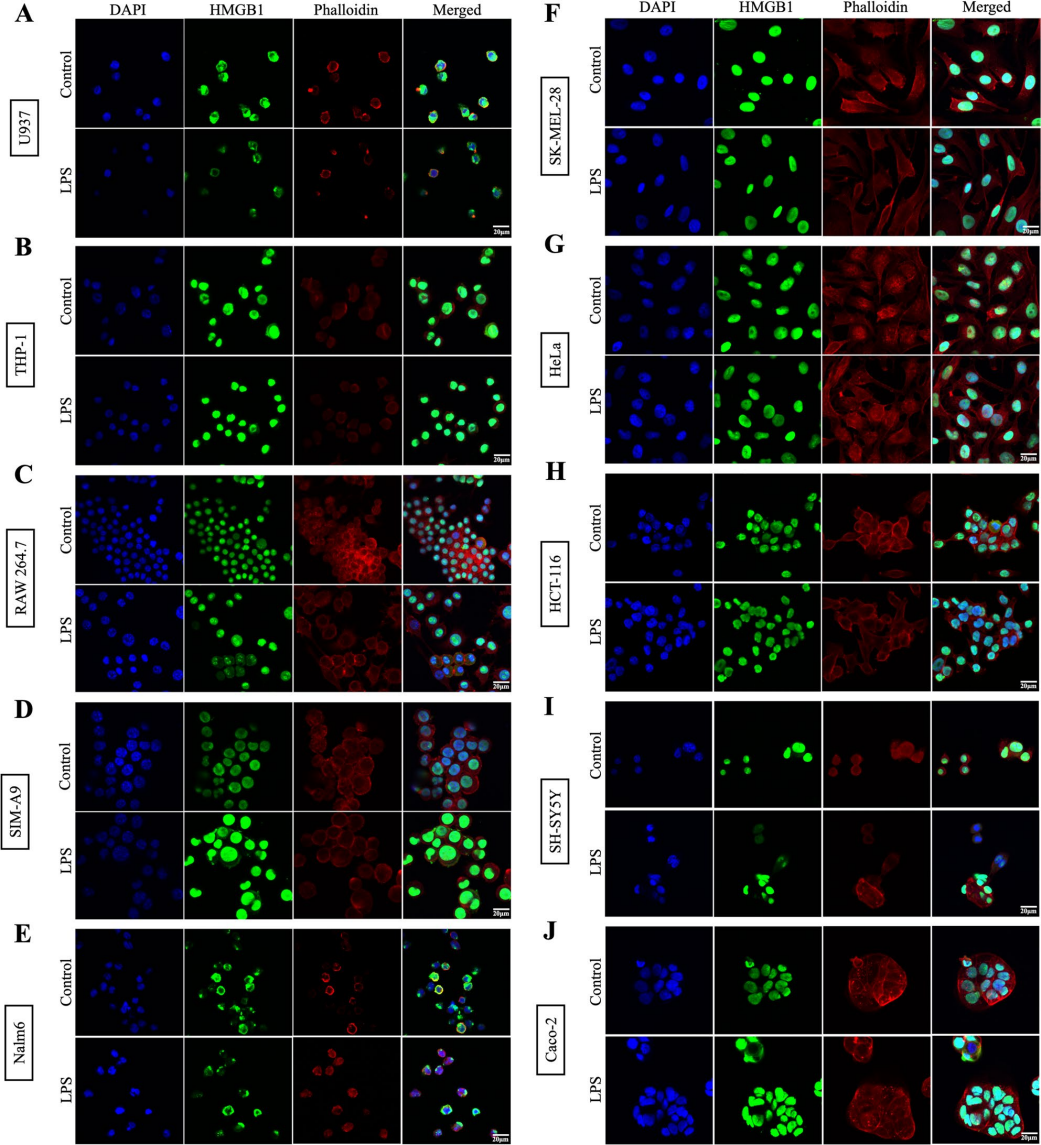
HMGB1 localization under basal and LPS-treated conditions. Immunofluorescence staining of **(A)** U937, **(B)** THP-1, **(C)** RAW 264.7, **(D)** SIM-A9, **(E)** Nalm6, **(F)** SK-MEL-28, **(G)** HeLa, **(H)** HCT-116, **(I)** SH-SY5Y, and **(J)** Caco-2 cells under untreated and LPS-stimulated conditions. Cells were stained with DAPI (blue) to visualize nuclei, anti-HMGB1 monoclonal antibody (green) to detect HMGB1 localization, and phalloidin (red) to stain F-actin. Merged images show the overlay of all three stains. Scale bars represent 20 μm

To confirm the presence of HMGB1 in selected myeloid-like and tumour cell lines, and to assess its subcellular localization following LPS-induced stress, we performed immunofluorescence stainings on the myeloid-like U937, THP-1, RAW 264.7 and SIM-A9 cell lines, as well as the tumor cell lines Nalm6 (Fig. 2A–E), SK-MEL-28, HeLa, HCT-116, SH-SY5Y, and Caco-2 cells (Fig. 2F–J). Under basal conditions, HMGB1 was predominantly localized in the nucleus across most of the cell lines, as indicated by HMGB1-DAPI co-staining. U937 and Nalm6 were exceptions exhibiting low colocalization under both control and LPS conditions, consistent with constitutive cytoplasmic localization of HMGB1. After 24 h of LPS treatment, localization patterns diverged markedly between cell types. THP-1, HeLa, HCT-116, and Caco-2 cells largely retained nuclear HMGB1 with minimal change upon stimulation. In contrast, RAW 264.7, SK-MEL-28 and SH-SY5Y cells showed reduced nuclear colocalization following LPS treatment, suggesting partial cytoplasmic redistribution. SIM-A9 cells displayed variable colocalization under both conditions, without a consistent LPS-dependent pattern. Qualitative assessment of these images supported these observations of HMGB1-DAPI colocalization (Fig. S1). Overall, these findings indicate that HMGB1 is constitutively expressed across the examined tumor and immune cell lines with different localization patterns.

### HMGB1 is released as monomers or in complexes following cell death

Cell death is an intrinsic feature for both tumour cells and infiltrating immune cells in the tumour microenvironment. To investigate whether the mode of cell death influences the release of HMGB1, we induced necrosis, apoptosis, and pyroptosis in myeloid THP-1 cells and examined proteins derived from culture supernatants with Western blot. We opted to use THP-1 cells due to their responsiveness to pyroptotic stimuli, thus providing a robust model for comparing HMGB1 dynamics across distinct death programs. LDH (necrosis), Caspase 3/7 activity (apoptosis), and IL-1β secretion (pyroptosis) were used to confirm successful induction of the respective cell deaths (Fig. S2).

**Fig. 3:**
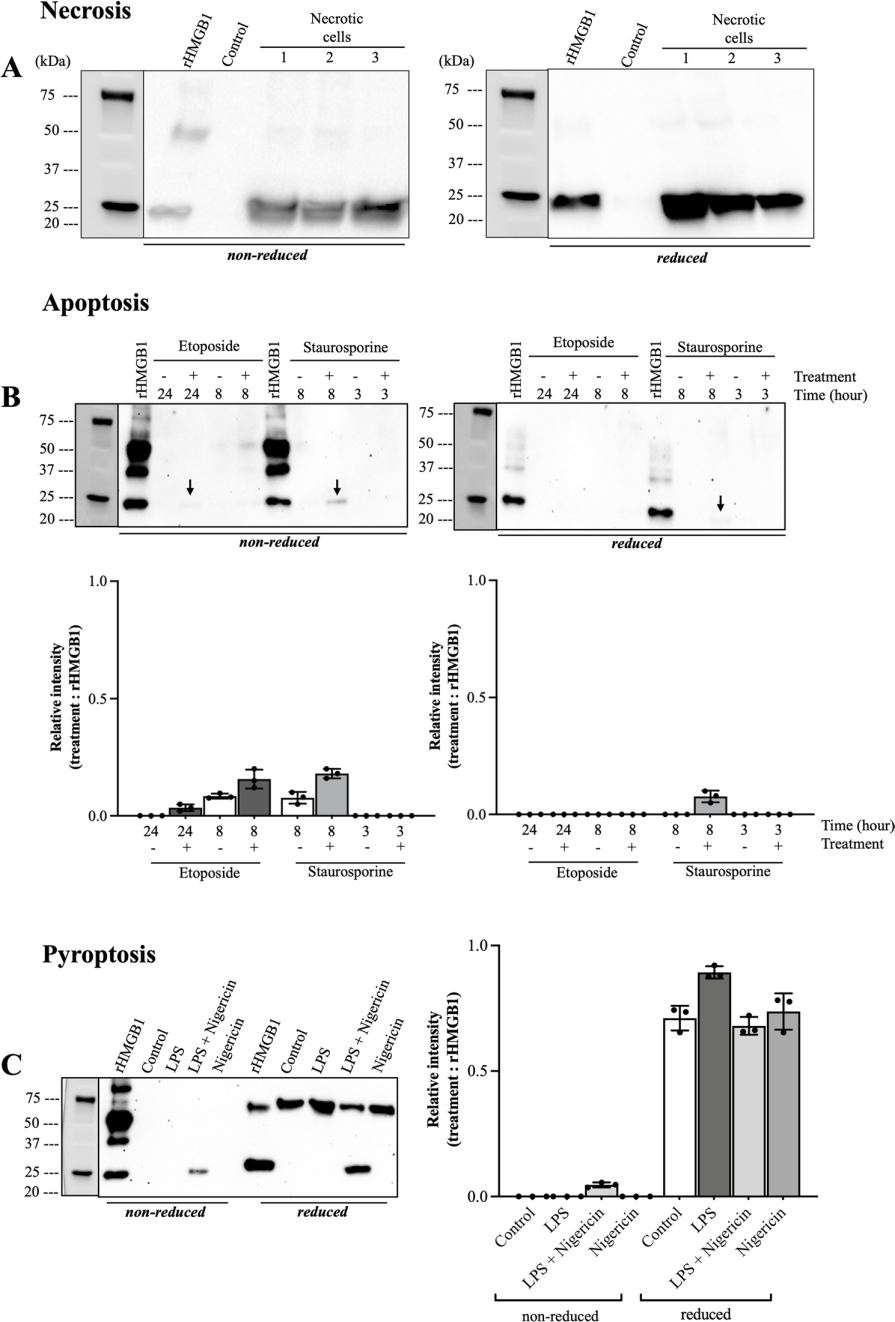
HMGB1 monomers and complexes are formed depending on the mode of cell death. Western blotting under reducing and non-reducing conditions was used to detect HMGB1 in TCA-precipitated protein extracts from culture supernatants of **(A)** control (*n* = 1), necrotic THP-1 cells (*n* = 3), **(B)** etoposide- or STS-treated apoptotic THP-1 cells (*n* = 3), and **(C)** PMA-differentiated, LPS- nigericin-treated pyroptotic THP-1 cells (*n* = 3). Blots are representative of three independent experiments. Relative HMGB1 band intensities were quantified. [rHMGB1 – all bands in recombinant HMGB1 lane used as normalizing control, nd = not detected]

A band corresponding to monomeric HMGB1 at 25 kDa was observed in Western blots with proteins from necrotic cell supernatants (Fig. 3A) under both reducing and non-reducing conditions. No band at 50 kDa that could correspond to dimeric HMGB1 was detected under non-reducing conditions. Western blot in non-reducing conditions of proteins from apoptotic THP-1 cell supernatants showed faint bands corresponding to monomeric HMGB1 at 25 kDa for both etoposide (24 h incubation) and STS (8-hours incubation) treated cells (Fig. 3B). Also, faint 50 kDa bands were observed in Western blots from 8-hour etoposide-treated cell cultures. However, it cannot be excluded that this signal is a potential attribute of overspill from the neighboring rHMGB1 control lane. Under reducing conditions, no HMGB1 bands were detected at any time point in etoposide-treated cell supernatants (Fig. 3B). For 8-hour STS-treated cell supernatants, a 25 kDa HMGB1 band was observed under non-reducing conditions, whereas only a faint band slightly below 25 kDa was seen under reducing conditions. The shift in size could be caused by partial loss of the c-tail part of the molecule, a feature known to occur for rHMGB1 and also evident in Fig. 3A and B. No HMGB1 bands were detected in supernatants from untreated cells or from cells treated with STS for 3 h.

To investigate HMGB1 release during pyroptosis, PMA-differentiated macrophage-like THP-1 cells were primed with LPS, followed by treatment with nigericin, a potassium ionophore and NLRP3 inflammasome inducer (Evavold et al. 2018). Under non-reducing conditions, an HMGB1 band at 25 kDa was observed in supernatants of pyroptotic (LPS + Nigericin stimulated) cells but not in any of the other conditions (Fig. 3C). Interestingly, upon protein reduction with TCEP, strong bands above 50 kDa appeared for all culture conditions and the 25 kDa band in LPS+nigericin treated cell supernatants became more intense than in non-reducing conditions. This suggests a redox-dependent release of monomeric HMGB1 from protein complexes. The appearance of high molecular weight bands during reducing conditions indicates HMGB1-containing complexes that are not formed by disulfide bonds. Although the reduction of the ∼50 kDa band in the LPS + nigericin condition, accompanied by an increase of the 25 kDa band might suggest the presence of disulfide-linked species, this pattern is also consistent with stimulus-dependent changes in heterocomplex composition. No stable extracellular HMGB1 homodimers were detected under the non-reducing conditions examined, though low-abundance or transient homo-dimeric HMGB1 cannot be formally excluded. It also suggests that the epitope recognized by the antibody used for Western blotting is hidden during non-reducing conditions. Taken together, these results demonstrate that HMGB1 release from THP-1 cells is influenced by the mode of cell death, with necrosis and pyroptosis leading to detectable monomeric HMGB1 in supernatants, while apoptosis results in lower and more variable release. Additionally, PMA-differentiated THP-1 cells release redox-independent HMGB1 hetero-complexes, whereas monomeric HMGB1 is only released upon pyroptosis.

### HMGB1 is released as monomers or complexes by myeloid and tumour cell lines

**Fig. 4:**
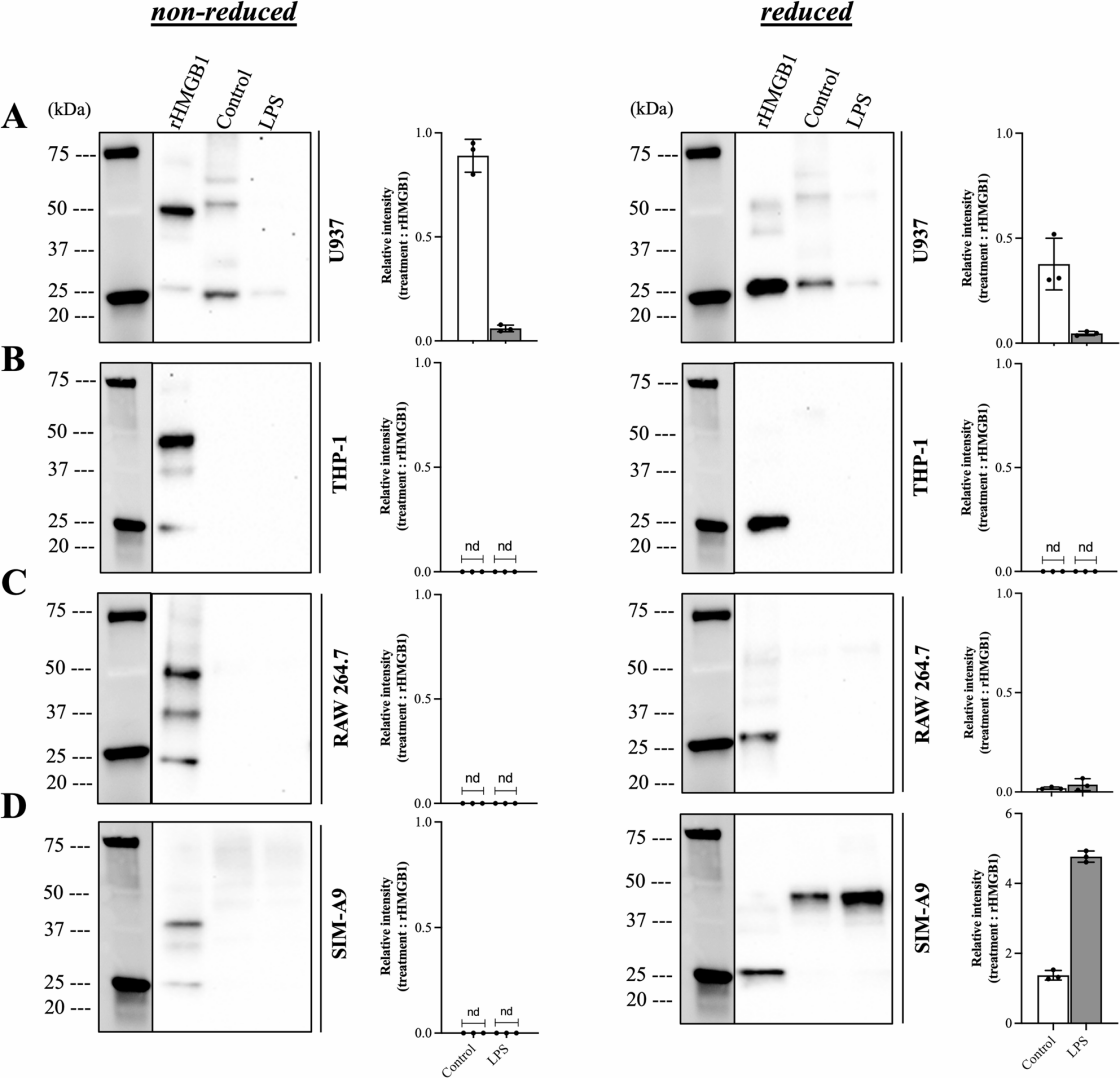
HMGB1 secretion is impacted by LPS stress in myeloid tumour cell lines U937 and SIM-A9 cells. The left panel displays western blots of HMGB1 from myeloid-derived cell line supernatants. The right panels show the corresponding band intensity analysis of HMGB1 (normalized to rHMGB1) for each condition. Error bars represent mean ± SD from three independent experiments. One representative blot image is shown here from three independent replicates. [rHMGB1 – all bands in recombinant HMGB1 lane used as normalizing control, nd = not detected]

Given that myeloid and tumour cells are known to actively secrete HMGB1 and often exhibit high intracellular oxidative conditions (Gardella et al. 2002, Tang et al. 2011), we next assessed whether the cells of myeloid tumour lineages release dimeric HMGB1 upon LPS-induced stress. To control for passive HMGB1 release due to cell death with membrane rupture, cytotoxicity was measured using an LDH assay. Across all cell lines, cell death ranged from 2% to 15% in both untreated and LPS-treated conditions (Fig. S3). Analysis of cell supernatants from unstimulated promonocytic U937 cells showed strong bands at 25 kDa (Fig. 4A) in both non-reducing and reducing conditions, while LPS stress resulted in faint bands. Similarly, weaker bands at 50 kDa were observed for unstimulated cell supernatants that were only faintly visible after LPS treatment. These bands remained under reducing conditions, implicating HMGB1 redox-independent heterocomplexes rather than redox-dependent HMGB1 homodimers. In contrast, no HMGB1 was detected when analyzing mouse macrophage-like RAW 264.7 or monocytic THP-1 cell supernatants, irrespective of non-reducing or reducing conditions (Fig. 4B, C). In blots of mouse microglial SIM-A9 cell supernatants, strong bands at 50 kDa were observed for both unstimulated and LPS-stimulated cells under reducing conditions (Fig. 4D).

**Fig. 5:**
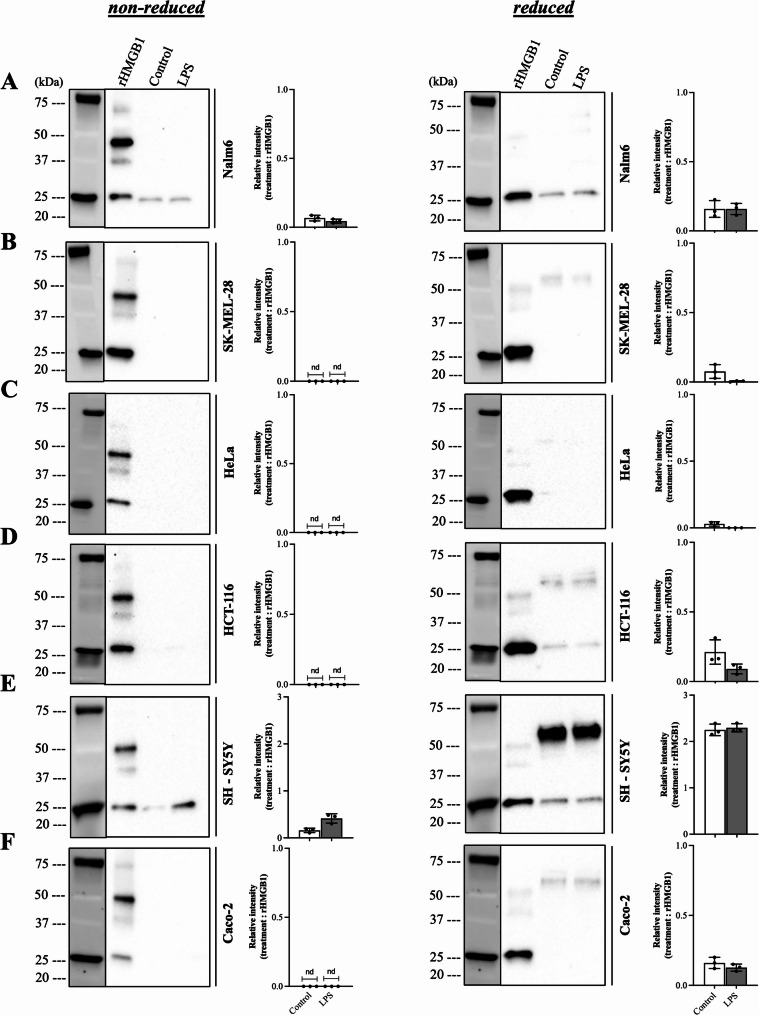
HMGB1 is released as a monomer or in complexes in a set of tumour cell lines, irrespective of LPS stress. The left panel displays Western blots of HMGB1 in supernatants from the investigated tumour cell lines. The right panels show the corresponding band intensity analysis of HMGB1 (normalized to rHMGB1) for each condition. Error bars represent mean ± SD from three independent experiments. One representative blot is shown for three independent replicates. [rHMGB1 – all bands in recombinant HMGB1 lane used as normalizing control, nd = not detected]

In the investigated tumour cell lines of different non-myeloid lineages, bands at 25 kDa were observed in both lymphocytic Nalm6 and neuroblastoma SH-SY5Y cell supernatants under non-reducing and reducing conditions, and for colorectal carcinoma HCT-116 cell supernatants under reducing conditions. Strong bands of a molecular weight higher than 50 kDa appeared only in reduced SH-SY5Y samples and were not detected in Nalm6 under any condition (Fig. 5A, E). The intensity of the bands was not affected by LPS stress, and their molecular weight and presence in reducing conditions suggest that they are HMGB1 heterocomplexes. Interestingly, similar faint bands with a molecular weight higher than 50 kDa were observed in blots from reduced supernatant samples of melanoma SK-MEL-28 cells, the uterus cancer cells HeLa, colorectal carcinoma HCT-116 cells, and intestinal epithelial adenocarcinoma Caco-2 cells (Fig. 5B, C, D, and F).

### HMGB1 released by SIM-A9 cells interacts with ribosomal proteins, histone H2B and SRP9

Western blotting under reducing conditions revealed bands at 50 to 60 kDa in protein extracts from cell culture supernatants from SIM-A9, SK-MEL-28, HeLa, HCT-116, SH-SY5Y, and Caco-2 cell lines (Fig. 4A, D, and 5B-F). The persistence of such high molecular weight bands under reducing conditions implicates that HMGB1 is bound to other molecules independent of disulfide bridges. As such complexes were detected in both SIM-A9 and SH-SY5Y cells, but only showed LPS-dependent band intensity changes in SIM-A9 cells, we opted to further investigate their composition and identifying HMGB1-interacting partners in SIM-A9 cells using co-immunoprecipitation and LC-MS/MS. SIM-A9 cells were transduced to express p3xFlag-HMGB1 (SIM-A9^Flag+ HMGB1^) or non-tagged HMGB1 (SIM-A9^Flag− HMGB1^, negative control) were cultured with or without LPS, and mouse anti-FLAG^®^ M2 magnetic beads were used to isolate HMGB1 and its binding partners from culture media.

**Fig. 6:**
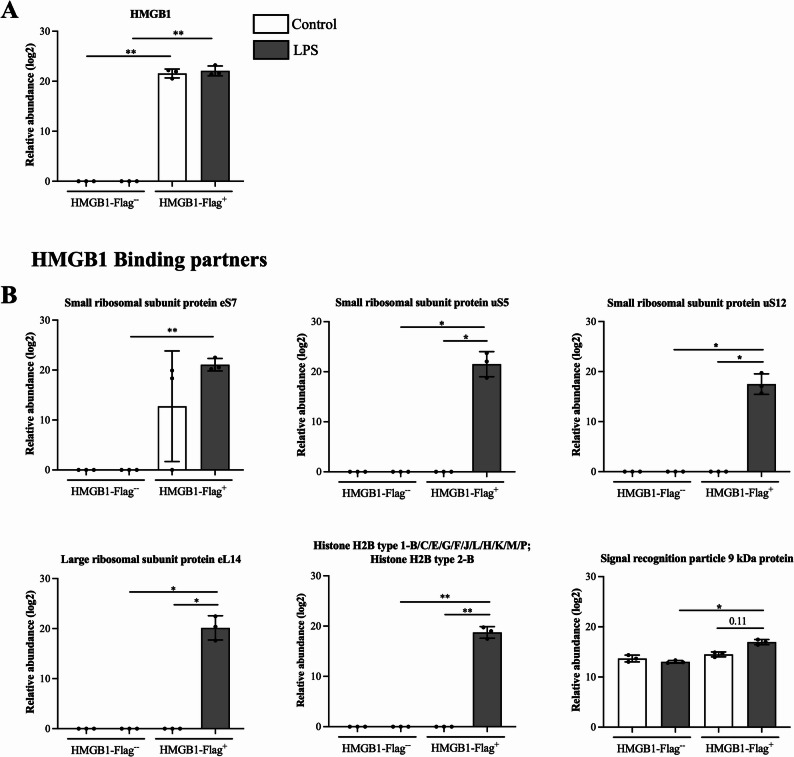
Released HMGB1 is in complex with ribosomal proteins, histone H2B, and SRP9. To identify HMGB1 binding proteins, SIM-A9 cells transduced to express p3xFlag-HMGB1 (SIM-A9^p3xFlag +HMGB1^) or non-flag HMGB1 (SIM-A9^p3xFlag −HMGB1^, negative control) and thereafter stimulated with 1 µg/ml LPS or control (PBS). Collected culture supernatants were incubated with anti-Flag M2 magnetic beads to purify p3xFlag-HMGB1 and any interacting proteins. Following co-immunoprecipitation, LC/MS-MS was used to identify eluted proteins and data is showing abundance of HMGB1 **(a)** and HMGB1 binding proteins **(b)**. Data presented as mean ± SD from three technical replicates (*n* = 3). Statistical analysis was done using two-sample t-tests comparing SIM-A9^p3xFlag+HMGB1^ versus SIM-A9^p3xFlag−HMGB1^ on log_2_ transformed relative values. P-values were corrected for multiple testing using Benjamini-Hochberg and q values of less than 0.05 were considered significant. * ≤ 0.05, ** ≤ 0.01

Flag-HMGB1 was successfully isolated from SIM-A9^p3xFlag−HMGB1^ cell culture supernatant (Fig. 6A). Interestingly, no difference in HMGB1 levels was found in supernatant from unstimulated and LPS-stimulated SIM-A9^p3xFlag−HMGB1^ cells. HMGB1 binding partners were defined as proteins found at significantly increased levels in supernatant from SIM-A9^p3xFlag+HMGB1^ compared to SIM-A9^p3xFlag−HMGB1^ (*p* ≤ 0.05). Notably, we identified five proteins to be isolated together with flag-HMGB1 following LPS treatment; small ribosomal subunit protein uS5, small ribosomal subunit protein uS12, large ribosomal subunit protein eL14, signal recognition particle 9 kDa protein (SRP9), and Histone H2B type 1-B/C/E/G/F/J/L/H/K/M/P; Histone H2B type 2-B. Only one protein was identified to interact with HMGB1 in both resting and LPS stimulated cells, namely Ribosomal subunit protein eS7 (Fig. 6B). While Flag-tag immunoprecipitation is highly selective, interactions with the Flag-tag itself cannot be entirely excluded, the identified proteins should be considered as putative HMGB1-associated factors rather than definitely confirmed binding partners.

## Discussion

HMGB1 contributes to cancer pathogenesis, and increased expression has been linked to increased severity and poorer survival (Vladimirova et al. 2025). Therapeutic targeting of this molecule remains challenging as its effects vary depending on not only its oxidative status but also its structural form—monomeric, dimeric, or incorporated into heterocomplexes. The factors that determine the formation of these extracellular forms are not well understood. In this study, we examined HMGB1 release and forms of HMGB1 released both from multiple tumour cell lines and from myeloid cell lines to mimic cellular sources of HMGB1 in the tumour microenvironment. We also used LPS as a stressor to study active induction of HMGB1 release and induced different types of cell death, both processes associated with the tumour microenvironment. Our results demonstrated that HMGB1 is released as a monomer or in a complex with other molecules, with the pattern influenced by cell type, mode of cell death, and cellular activation state.

Consistent with previous reports, we recorded HMGB1 release from necrotic cells (Scaffidi et al. 2002) and we observed it as a monomeric 25 kDa protein. In contrast, HMGB1 release during apoptosis was limited. This is in agreement with a previous study showing that HMGB1 remains bound to chromatin in apoptotic cells (Bell et al. 2006). In LPS- and nigericin-induced pyroptosis, we detected HMGB1 released as a monomer. This is consistent with previous reports of inflammasome-dependent HMGB1 secretion during pyroptotic cell death (Lamkanfi et al. 2010, Kayagaki et al. 2015). Notably, under reducing conditions, Western blot revealed HMGB1 at > 50 kDa in all supernatants from macrophage-differentiated THP-1 cells, suggesting the formation of extracellular complexes with epitopes masked under non-reducing conditions and binding independent of disulfide bridges. Overall, these findings reveal a pronounced HMGB1 release during necrosis and pyroptosis whereas it remains limited during apoptosis. During both necrosis and pyroptosis, membrane integrity is lost, which allows for HMGB1 to be passively released as a monomer.

HMGB1 release from myeloid cells has previously been demonstrated to increase following LPS treatment (Lu et al. 2014). Our data reveal that HMGB1 release in response to LPS is cell type dependent. LPS treatment of pre-monocytic U937 cells leads to a decrease in HMGB1 release whereas LPS treatment of microglial SIM-A9 leads to an increase in HMGB1 release. However, no HMGB1 was detected in either resting or LPS treated monocytic THP-1 or macrophage-like RAW cells. Interestingly, U937 cells released HMGB1 detectable at various molecular weights by Western blotting while SIM-A9 cell supernatants contained protein complexes clearly visible as bands at 50 kDa. Among the additional investigated tumour cell lines, monomeric HMGB1 (25 kDa) was detected in Nalm6 and SH-SY5Y cell supernatants in non-reducing condition Western blots. This indicates a release of HMGB1 in its monomeric form. Interestingly, bands at 60 kDa appeared in Western blots under reducing conditions for supernatants from SK-MEL-28, HCT-116, SH-SY5Y and Caco-2 cell lines. The persistence of these higher molecular weight bands under reducing conditions suggests the formation of stable HMGB1-protein complexes rather than disulfide-linked HMGB1 dimers.

The heterogeneity in LPS-induced HMGB1 translocation and secretion across cell lines likely reflects both technical and biological factors. The investigated cell lines most likely vary in downstream signaling capacity and varies in TLR4 expression. Moreover, HMGB1 translocation is a modification-dependent process. These signaling events may progress with different kinetics depending on cell type. These biological differences, together with the detection of released HMGB1 accumulated during 24 h by western blotting and the imaged nuclear and cytoplasmic snapshot of HMGB1 localization after 24 h of LPS stimulation, can explain at the first glance disparate results from our Western blotting analysis and the immunocytochemistry performed.

Due to the persistent detection of non-reducible HMGB1 at a high molecular weight by Western blots, we screened for HMGB1 binding partners in supernatants from LPS-treated and untreated p3xFlag-HMGB1 expressing SIM-A9 cell cultures using mass spectrometry. Six HMGB1-interacting proteins were identified: histone H2B, signal recognition particle 9 (SRP9), and ribosomal proteins eS7, uS5, uS12, and eL14, of which five were increased in LPS-treated cells. Our findings on HMGB1 interacting with histone H2B are supported by studies showing that HMGB1 and histones are co-released extracellularly as components of neutrophil extracellular traps (NETs) (Tadie et al. 2013). HMGB1 can both induce NET formation and be released as part of the NET structure alongside DNA and histones, forming proinflammatory complexes that contribute to disease pathogenesis (Kim and Lee 2020, Magna and Pisetsky 2014). Interestingly, HMGB1-containing nucleosomes were previously found in Jurkat cell culture supernatant following induction of secondary necrosis after STS treatment, rather than following LPS-induced secretion as per our study (Urbonaviciute et al. 2008). HMGB1-containing nucleosomes have been shown to have potentiated proinflammatory properties and are speculated to contribute to the pathogenesis of diseases such as sepsis and autoimmune disorders; hence, targeting such HMGB1 complexes would be an attractive therapeutic strategy (Urbonaviciute et al. 2008).

We have previously reported that intracellular HMGB1 interacts with SRP9 in both resting and LPS-treated THP-1 cells (Heinbäck et al. 2025). The SRP complex is a ribonucleoprotein mediating translation to the endoplasmic reticulum. However, SRP9 has been reported to be present in exosomes (Berger et al. 2014), similar to HMGB1 (Gardella et al. 2002). Our data indicate that extracellular HMGB1 interacts with SRP9 following LPS activation, potentially because of their joint release via exosomes.

This study was initiated partly based on the findings by Kwak et al. (Kwak et al. 2021) that HMGB1 dimers could be detected in serum of mice with LPS-induced endotoxemia. In contrast, in none of our in vitro cell cultures could we detect HMGB1 dimers. This disparity might be explained by the more reductionistic milieu created by the in vitro cell culture methods and the intricate inflammatory milieu that exists in vivo during sepsis. Although LPS stimulation causes a strong inflammatory response in vitro, it might not accurately reflect the extent or duration of oxidative stress seen in vivo. Future research should investigate if mechanisms like hypoxia, exogenous ROS regulation or cytotoxic stress, which more closely resemble the oxidative environment of sepsis, induce dimer formation in vitro.

Our research has several limitations and highlights some of the difficulties when studying HMGB1. First, we did not define the redox isoforms of released HMGB1. At present, there is no readily available method for such analysis. Secondly, we had to concentrate the cell supernatants to detect HMGB1 by Western blotting. We opted to perform TCA precipitation as a well-established method suitable for handling of multiple samples. It is possible that this choice of method could affect our results, however, a comparative analysis of the patterns obtained by TCA precipitation and by centrifugal concentration devices of cell supernatants demonstrated qualitative similar results. Thirdly, this study is performed using in vitro cultures of cell lines and not by investigating tumour tissue specimens. There are no commercial antibodies available that can distinguish the different HMGB1 redox isoforms by immunochemistry, and whether available ELISAs equally detects all HMGB1 redox isoforms, monomeric and dimeric HMGB1, as well as HMGB1 heterocomplexes, is also unknown. For certain cell lines used in our study, HMGB1 could only be detected by Western blot following disulfide bond reduction with the reducing agent TCEP. This suggests the presence of stable HMGB1 complexes with epitopes masked from detection antibodies under non-reducing conditions. Non-covalent interactions, rather than disulfide bonds, especially salt bridges between the positively charged HMG box domains and the acidic tail of HMGB1, which are insensitive to reducing agents, are probably responsible for these complexes’ stability under reducing circumstances. Since ribosomal proteins are typically basic and would be expected to generate electrostatic interactions with the thirty-residue acidic tail of HMGB1, this is consistent with our found binding partners. However, while our study is limited by in vitro conditions, it provides a foundation for investigating HMGB1 structural dynamics in more complex settings. The biological significance of these heterocomplexes calls for additional research beyond structural characterisation. The ribosomal protein and histone H2B complexes found here may influence receptor engagement and inflammatory signalling inside the tumour microenvironment in a manner similar to well-known HMGB1 complexes like HMGB1/CXCL12, which increases inflammatory cell recruitment via CXCR4.

## Conclusion

Taken together, our results from the in vitro cell culture systems used show that HMGB1 release differs depending on cell type, mode of cell death and on cellular stress. HMGB1 was found to be released as monomers or in protein complexes, but no evidence of dimerized HMGB1 release was observed. This contrasts with previously reported findings of HMGB1 dimers in cell lysates treated with CuCl_2_/H_2_O_2_ or in serum from LPS-challenged mice (Kwak et al. 2021). Our co-immunoprecipitation results revealed that released HMGB1 formed stable complexes with numerous proteins, in particular with ribosomal subunit proteins. Furthermore, HMGB1 binding partners differed following LPS challenge, suggesting that HMGB1 may participate in distinct extracellular protein networks depending on cellular activation states. Our data sheds light molecular aspects of the complex biology of HMGB1, with implications for its divergent roles in cancer pathology.

## Supplementary Information

Supplementary Material 1.

Supplementary Material 2.

## Data Availability

The mass spectrometry proteomics data have been deposited to the ProteomeXchange Consortium via the PRIDE (Perez-Riverol et al. 2025) partner repository with the dataset identifier PXD077444. All other data supporting the findings of this study are included within the article and its supplementary information.
