# Supplementary material for: Context-dependent release of HMGB1: cell death mode, cell type and LPS stress drive monomer and heterocomplex formation
Source: Mol Med. 2026 May 2;32:93. doi: 10.1186/s10020-026-01489-2 (PMC13281463; doi:10.1186/s10020-026-01489-2)
Supplement: Supplementary file 1 — Supplementary Material 1. [file 10020_2026_1489_MOESM1_ESM.docx]

**Supplementary table. 1. Cell lines and culture media used in this study.**

| **Cell line** | **Catalog number** | **Cell type** | **Culturing media** |
| --- | --- | --- | --- |
| U937 | CRL-1593.2 | Human histiocytic lymphoma (monocytic) | RPMI with 10% FCS, 100U/ml Penicillin and 0.1mg/ml Streptomycin |
| Nalm6 | CRL-3273 | Human pre-B acute lymphoblastic leukemia |  |
| RAW 264.7 | TIB-71 | Mouse macrophages |  |
| THP-1 | TIB -202 | Human monocytic leukemia |  |
| SIM-A9 | CRL-3265 | Mouse microglial cell line (immortalized) | DMEM: F12 with 10% FCS,  5% Horse serum, 100U/ml Penicillin and 0.1mg/ml Streptomycin |
| SH-SY5Y | CRL-2266 | Human neuroblastoma |  |
| HeLa | CCL-2 | Human cervical adenocarcinoma | DMEM with 10% FCS, 100U/ml Penicillin and 0.1mg/ml Streptomycin |
| HCT-116 | CCL-247 | Human colorectal carcinoma |  |
| Caco-2 | HTB-37 | Colorectal adenocarcinoma |  |
| SK-MEL-28 | HTB-72 | Human malignant melanoma |  |
