# Supplementary material for: Context-dependent release of HMGB1: cell death mode, cell type and LPS stress drive monomer and heterocomplex formation
Source: Mol Med. 2026 May 2;32:93. doi: 10.1186/s10020-026-01489-2 (PMC13281463; doi:10.1186/s10020-026-01489-2)
Supplement: Supplementary file 2 — Supplementary Material 2. [file 10020_2026_1489_MOESM2_ESM.docx]

**Supplementary Figures**

Control

LPS

LPS + IFNγ

**
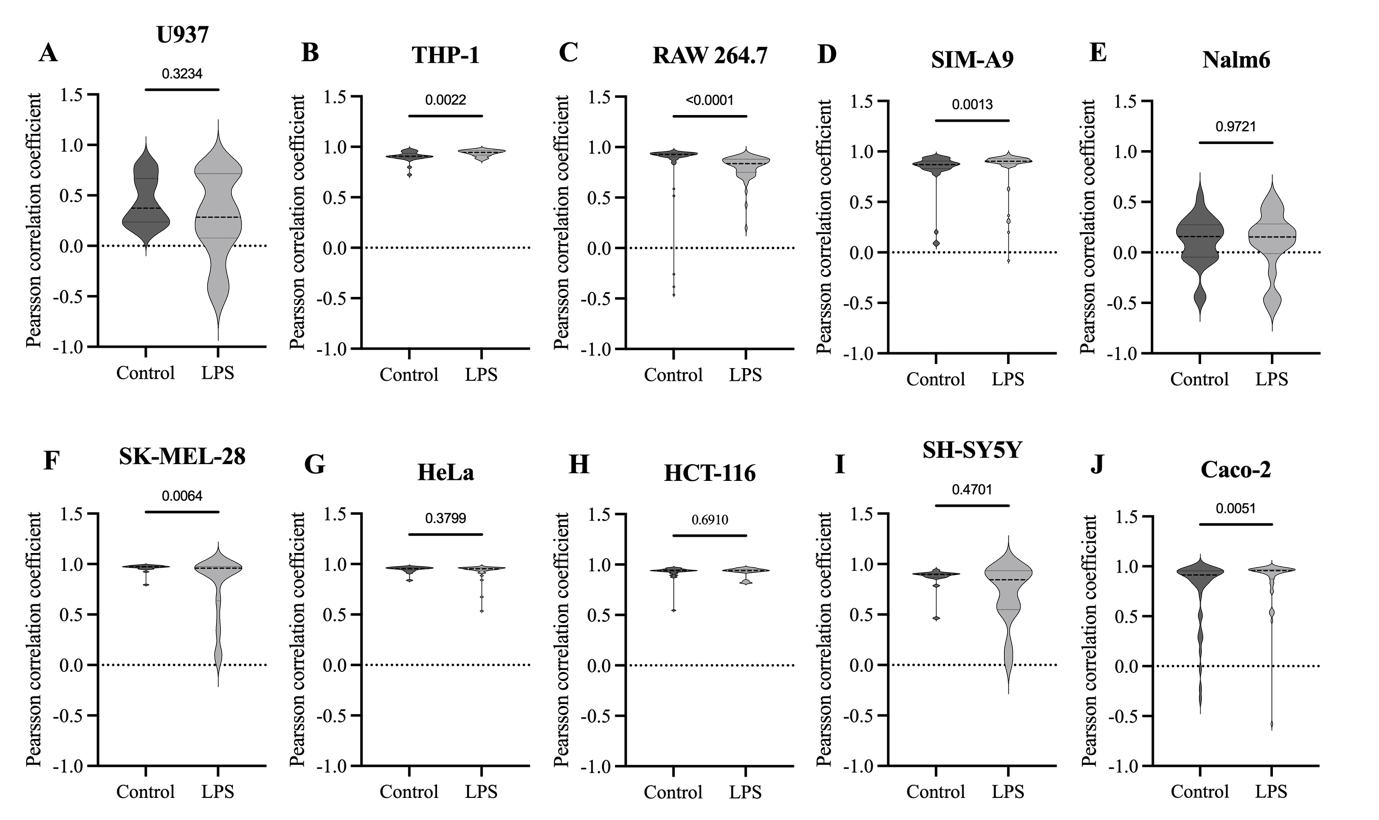
**

**Figure S1. Qualitative assessment of HMGB1 nuclear colocalization in tumour cell lines following LPS stimulation.** ICC analysis of HMGB1 distribution under control and LPS-stimulated (1 µg/ml for 24 hours) conditions. Pearson’s correlation coefficient was used to assess colocalization between the protein of interest and a nuclear marker. Values close to 1 indicates strong nuclear localization, while a decrease reflects translocation to the cytoplasm. Violin plots depict the distribution of single-cell measurements. Statistical analysis was performed using the Mann-Whitney test for pairwise comparisons, with exact p-values indicated on the graph.

**
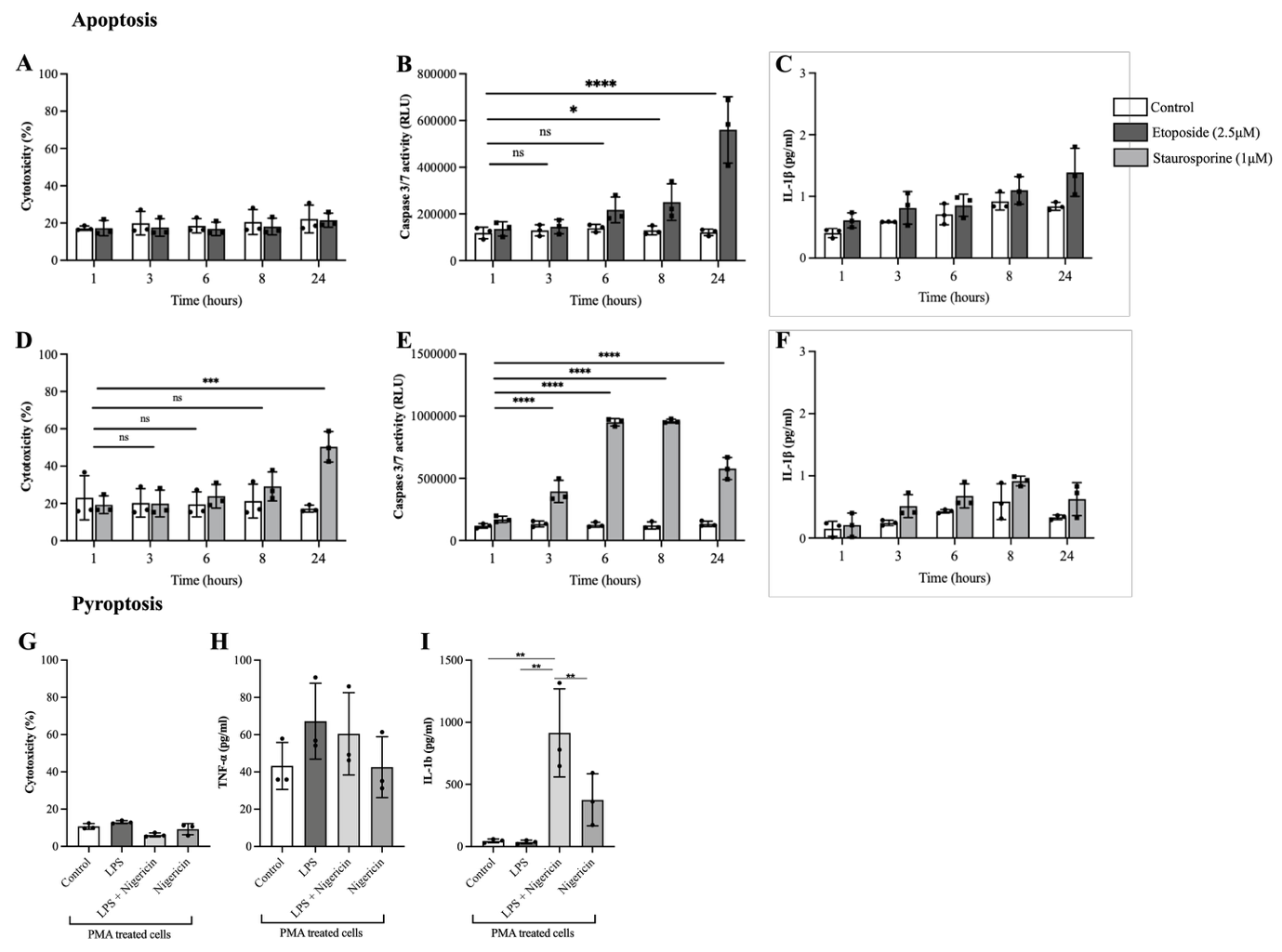
**

**Figure S2. Verification assays for assessing the induction of different cell deaths. Apoptosis:** THP-1 cells were treated with etoposide (2.5μM) or staurosporine (1 μM) for 1 to 24 hours to induce apoptosis. Supernatants were collected and analyzed for **(A, D)** LDH release to determine the cytotoxicity. Tx-100-treated cells were used as 100% lysed cell controls. **(B, C)** Caspase 3/7 activity presented as relative luminescence units (RLU) and **(C, F)** IL-1β release**. Pyroptosis:** THP-1 cells differentiated into macrophage-like cells by PMA (100 ng/ml) were primed with LPS (10 ng/ml) and treated with Nigericin (10 μM) to induce pyroptosis. **I)** LDH, **J)** TNF-α, and **K)** IL-1β release were measured in cell supernatants. Data presented is from three independent experiments (n=3) (mean ± SD). DMSO and EtOH was used as a negative control for apoptosis; Two-way ANOVA was performed for ELISA, *p < 0.05, **p<0.01, ***p < 0.001, and ****p < 0.0001.


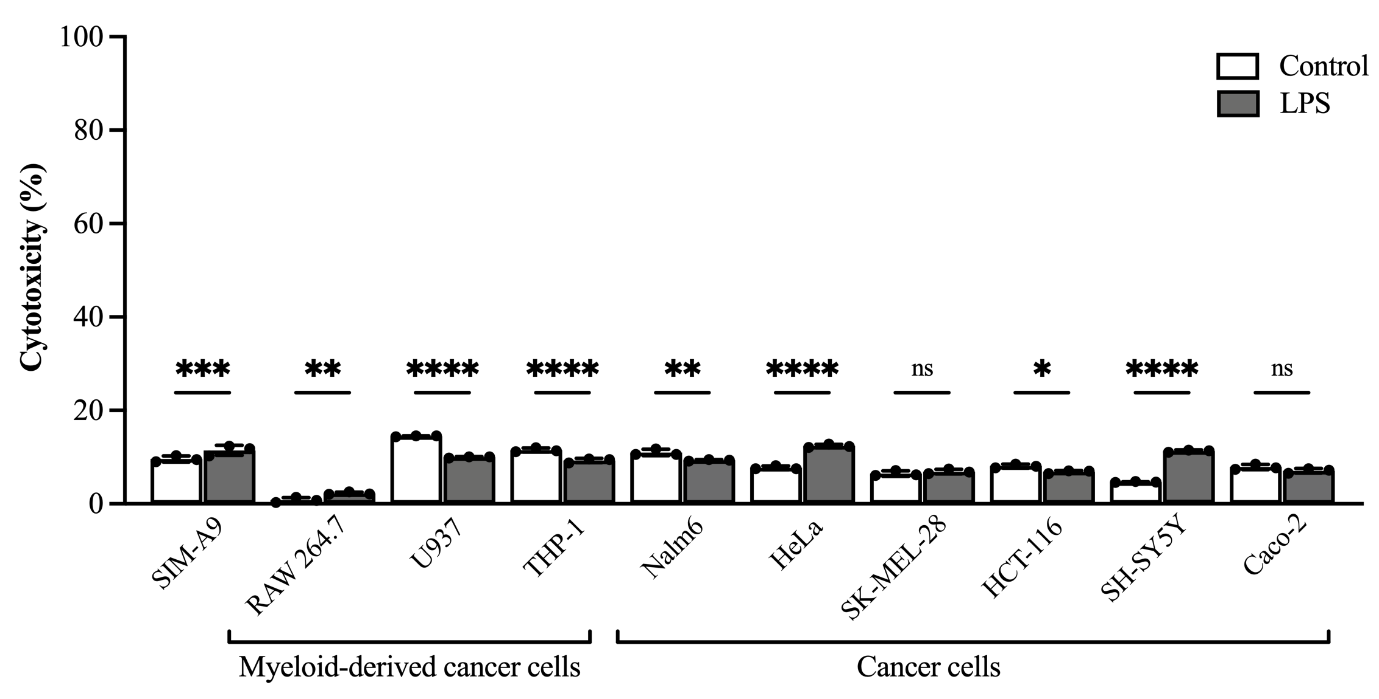
**Figure S3. Cytotoxicity assessment of tumour cell lines following LPS treatment using LDH release assay.** Cytotoxicity was evaluated in various tumour cell lines using an LDH release assay. The percentage of cytotoxicity was calculated relative to a 100% cell death control (2% Triton X-100). Cell lines are grouped into myeloid-derived and other tumour types. Data are presented as mean ± SD from three independent replicates. Statistical analysis was performed using two-way ANOVA and p values were corrected for multiple testing using Šidák correction. *p < 0.05, **p < 0.01, ***p < 0.001, and ****p < 0.0001.
